# Minor Salivary Gland Carcinosarcoma of the Floor of the Mouth

**DOI:** 10.7759/cureus.37206

**Published:** 2023-04-06

**Authors:** Timothy B Shaver, Emily Youner, Joseph F Goodman, Shabnam Samankan

**Affiliations:** 1 Otolaryngology - Head and Neck Surgery, George Washington University School of Medicine and Health Sciences, Washington, USA; 2 Pathology, George Washington University School of Medicine and Health Sciences, Washington, USA

**Keywords:** floor of the mouth, cancer, pathology, head and neck, minor salivary gland, carcinosarcoma

## Abstract

Carcinosarcoma is an uncommon tumor consisting of malignant epithelial and mesenchymal elements. Salivary gland carcinosarcoma is aggressive in nature, and given its biphasic histologic appearance, it has the potential to be mistaken for a less concerning entity. Intraoral minor salivary gland carcinosarcoma is exceedingly rare with the palate being the site most frequently involved. Only two cases of carcinosarcoma arising from the floor of the mouth (FOM) have been reported. We present a case of a non-healing FOM ulcer that was identified as a minor salivary gland carcinosarcoma on surgical pathology along with the steps and importance of accurate diagnosis.

## Introduction

Carcinosarcoma, also known as a true malignant mixed tumor, is a rare tumor composed of both malignant epithelial and mesenchymal elements. It represents less than 0.2% of all mixed salivary gland tumors [1]. Malignant mixed tumors may arise in the background of a preexisting pleomorphic adenoma (carcinoma ex pleomorphic adenoma and benign metastasizing pleomorphic adenoma) or less commonly may occur de novo as carcinosarcoma [1-3]. The parotid gland is the most commonly affected salivary gland, whereas the hard palate is the most common site in the oral cavity [4,5]. Intraoral carcinosarcoma involving the floor of the mouth (FOM) is exceedingly rare with only two cases have been reported in the literature [6,7]. We present a case of minor salivary gland carcinosarcoma of the floor of the mouth, in which the patient had no evidence of coexisting or preexisting pleomorphic adenoma.

## Case presentation

**Figure 1:**
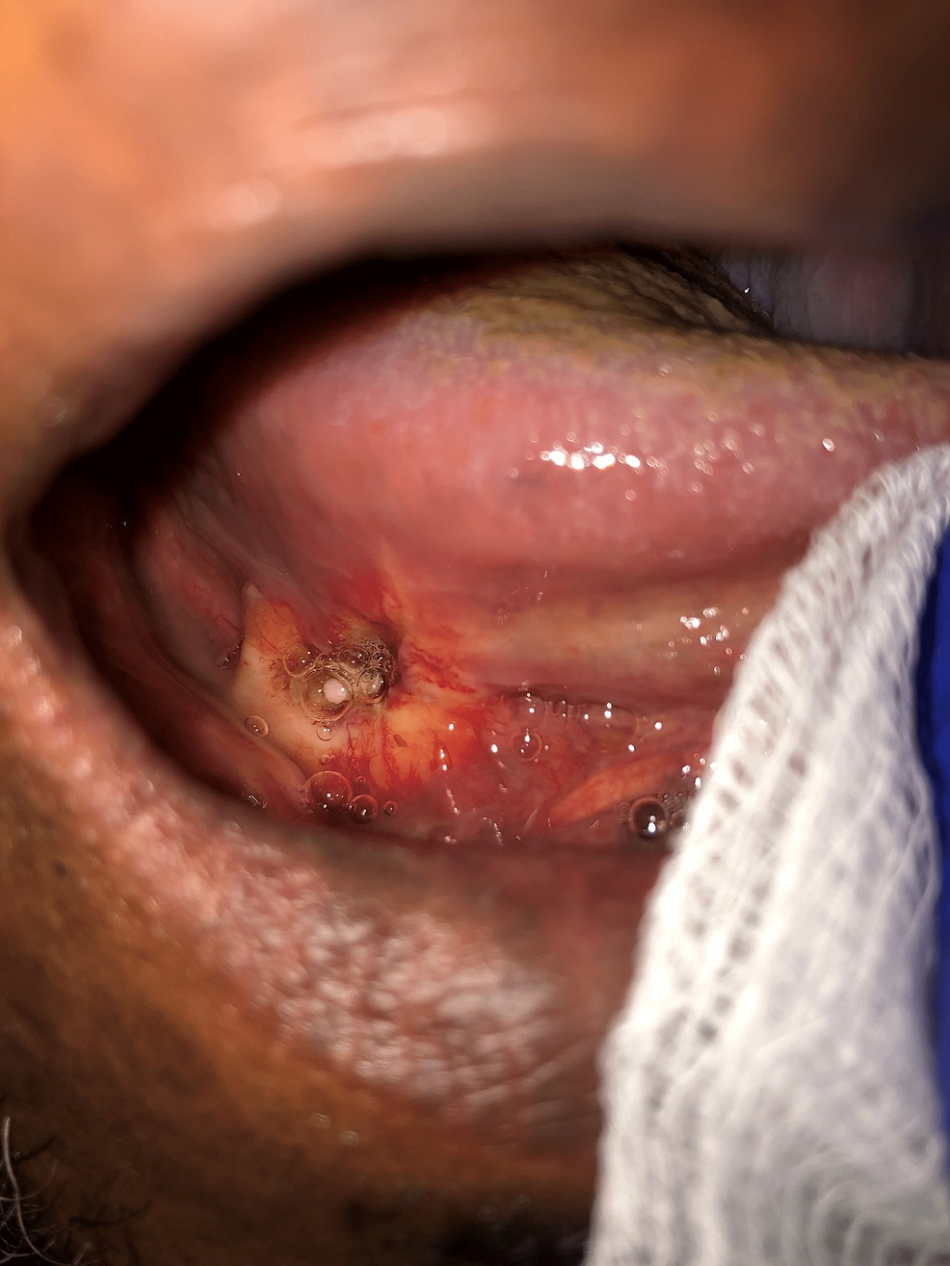
Ulcerative lesion at the junction of the right floor of the mouth/ventral tongue corresponding to patient's known malignancy.

A 67-year-old male presented to an otolaryngology clinic due to a five-month history of a right FOM ulcer. The patient had undergone an initial workup for this lesion at an outside facility where an excisional biopsy showed a poorly differentiated and high-grade adenocarcinoma. A thorough examination revealed a 2.0 cm shallow lesion involving the right FOM and ventral tongue (Figure 1).

A PET/CT scan following injection of 11.9 mCi of 18-fluoro-2-deoxyglucose (FDG) identified a hypermetabolic focus within the right tongue but no evidence of hypermetabolic activity within the neck or elsewhere. The patient underwent right-sided composite resection with neck dissection, which revealed ulcerated mucosa with an underlying relatively well-circumscribed, firm tumor intraoperatively. Further pathological examination of the surgical specimen contained a 2.6 cm infiltrative submucosal neoplasm centered under focally ulcerated squamous mucosa. Histologically negative margins were achieved. Examination of ipsilateral neck dissection revealed one involved lymph node.

**Figure 2:**
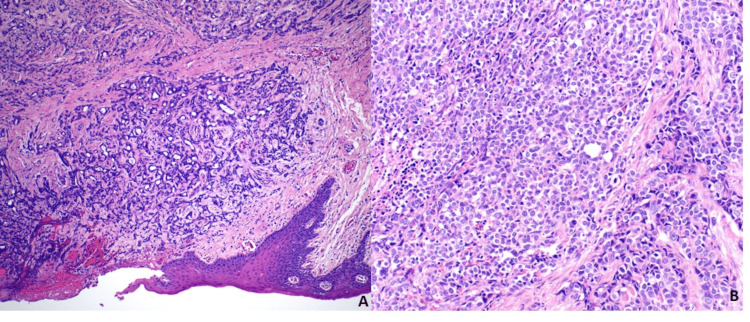
Malignant epithelial component arranged in a sheet-like glandular pattern. The images show (A) high-grade adenocarcinoma infiltrating beneath the ulcerated squamous mucosa (H&E, ×20) and (B) sheets of basaloid cells with marked pleomorphism, prominent nucleoli, abundant apoptotic cells, and mitotic figures. Clearing of cytoplasm is observed (H&E, ×200).

**Figure 3:**
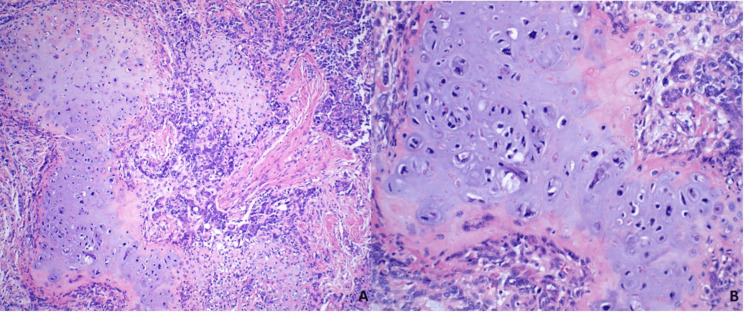
Malignant mesenchymal component. The images show (A) low-magnification chondrosarcoma surrounded by sheets of poorly differentiated carcinoma (H&E, ×40) and (B) high-magnification chondrosarcoma with atypical nuclei (H&E, ×200).

Microscopically, the tumor showed a biphasic differentiation. The epithelial component was predominantly composed of poorly differentiated adenocarcinoma comprising nests, ductules, and sheets of round basaloid cells with cytoplasm that varied from sparse to abundant cytoplasmic clearing (Figures 2A, 2B). Intermixed within the carcinoma were scattered chondrosarcomatous foci ​​characterized by a chondroid matrix and atypical nuclei (Figures 3A, 3B).

**Figure 4:**
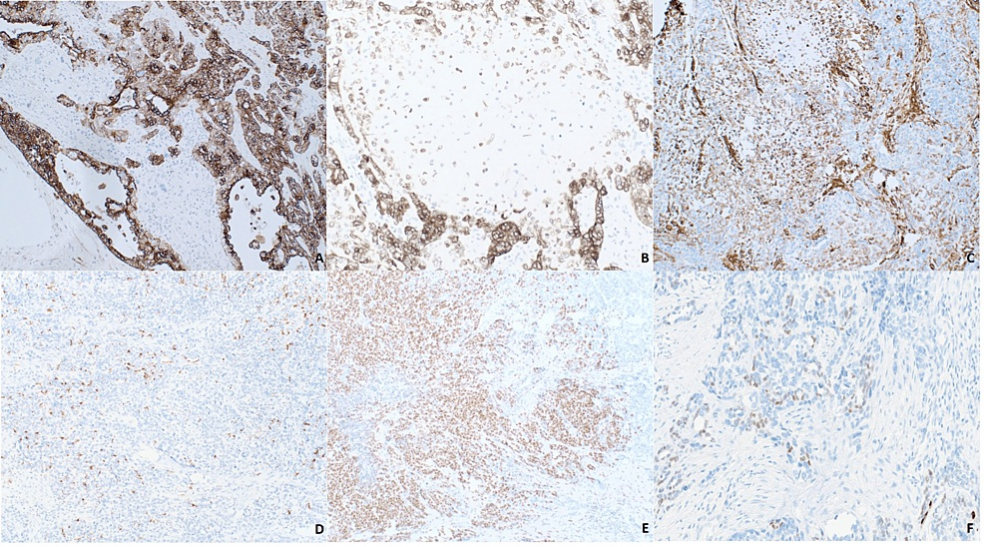
Immunohistochemical staining. The images show (A) diffuse AE1/AE3 immunostain highlighting carcinomatous component (×40), (B) scant weak AE1/AE3 expression by mesenchymal component (×200), (C) vimentin positivity by malignant chondroid cells and lack of reactivity in surrounding carcinoma (×200), (D) scattered S-100 protein-positive cells (×200), (E) nuclear androgen receptor (AR) expression (×200), and (F) weak and scant nuclear SOX10 expression (×400).

Histological features of salivary duct carcinoma were not present. Immunostaining demonstrated that the carcinomatous component diffusely expressed cytokeratin AE1/AE3 and CK7. Notably, a few cytokeratin-positive cells were identified in the sarcomatous stroma (Figures 4A, 4B). Vimentin was exclusive to chondroid areas (Figure 4C) while S100 and p63 positivity was scattered within the carcinoma but negative in the sarcomatous area (Figure 4D). Focal expression of androgen receptor (AR) was present, along with weak and patchy expression of SOX10, making the diagnosis compatible with salivary ductal carcinoma (Figures 4E, 4F). The tumor was negative for HER2(neu) by immunohistochemistry and fluorescence in situ hybridization (FISH). There was no evidence of coexisting or preexisting pleomorphic adenoma. The malignant tumor components were analyzed at the molecular level. RUNX1c.1183C>G is reported as a variant of unknown significance with an allelic variation of 48.0% (performed by hybrid capture next-generation sequencing {NGS} of tumor DNA, Tempus Laboratories, Chicago, IL). RNA sequencing revealed overexpression of the following genes: ERBB3, FGFR2, FGFR1, ERBB2 (HER2), CDKN2A, AR, and NY-ESO-1. The final pathologic diagnosis was de novo carcinosarcoma of salivary gland origin, as determined by the presence of two histologically and immunophenotypically distinct populations of neoplastic cells.

## Discussion

Carcinosarcoma is an exceedingly rare tumor of the salivary gland and carcinosarcomas of oral minor salivary glands are even rarer with only a few cases reported in the literature [1-8]. Carcinosarcoma regardless of location is a high-grade tumor having high rates of local recurrence [1]. Typically, poorly differentiated adenocarcinoma comprises the carcinomatous component, while the sarcomatous element is most frequently chondrosarcoma, followed by fibrosarcoma [8,9]. Should the sarcomatous component be the predominant tissue type; it is important to consider carcinosarcoma and search for definable carcinoma in such cases [3]. To date, only two cases of carcinosarcoma have been described in FOM. Both comprised of invasive squamous cell carcinoma and malignant spindle cell stroma but without documented myoepithelial characteristics [6,7]. This case, albeit similar to previous cases arising from FOM, is unique in its carcinomatous and sarcomatous components and immunophenotypical findings. The predominant tissue type was carcinomatous, and only scattered mesenchymal foci were identified. The former consisted of adenocarcinoma immunohistochemically consistent with salivary ductal carcinoma while the latter was composed of chondrosarcoma. It is still unclear whether the malignant constituents of salivary carcinosarcoma are independent of each other or if they are clonally related. It has been postulated that myoepithelial cells play a major role in the tumorigenesis of carcinosarcoma and may explain the coexpression of S-100, cytokeratin, and vimentin to different degrees in both constituents. Our findings are in keeping with previous views, scant chondroid cells were positive for cytokeratin and patchy S-100 was expressed throughout the tumor [8,9]. Neither of the previous cases originating from the FOM performed S-100 or other myoepithelial marker testing [6,7]. Due to the admixed nature of the biphasic malignant components, genotypic comparison between these morphologically distinct areas was not successful. There was no evidence of gene fusion/rearrangement by RNA sequencing. Allelic loss of TP53 gene had a high rate in our case, similar to the findings of Fowler et al. [10].

Salivary gland carcinosarcoma has a poor prognosis and carcinosarcoma de novo has even worse survival. Distant metastases, higher pT stage, and age are significantly associated with worse prognosis [11,12]. As carcinosarcoma is a rare entity, there is currently no therapeutic protocol that has been established as the gold standard. Several database studies have attempted to evaluate the prognostic factors and treatment options, however, most of the studies are focused on major salivary glands [2,11,12]. Treatment may consist of surgery alone or treatment combinations of surgery-radiotherapy and surgery-chemotherapy [8,12]. Anonsen et al. treated the patient with surgery, with no evidence of disease one year after surgery. Gallo et al. used combination therapy and the patient died after 10 months [6,7]. Our patient received surgical excision upfront, followed by adjuvant chemotherapy of weekly cisplatin (40 mg/m^2^) with six weeks of concurrent radiation therapy targeting the right FOM and ipsilateral neck. A PET/CT scan completed six months post-treatment identified mildly hypermetabolic irregularities around the surgical bed, thought to be post-treatment changes, but no evidence of gross residual or recurrent malignancy was seen.

## Conclusions

Although a rare entity, the aggressive nature of salivary gland carcinosarcoma and its potential to be mistaken for a less concerning lesion warrants more attention for prompt and accurate diagnosis. This case demonstrated a rare example of this uncommon entity originating from the FOM and the manner in which the diagnosis was made through stringent pathologic evaluation. In the context of multiple histologic elements, assuring proper diagnosis has clear implications for both prognosis and treatment.
